# Results From a Phase 4, Slow-Titration and Food Effect Trial to Assess the Safety and Efficacy of Xanomeline and Trospium Chloride in Schizophrenia

**DOI:** 10.1192/bjo.2026.11948

**Published:** 2026-06-30

**Authors:** Jenna Hoogerheyde, David Walling, Kimball Johnson, Rachel Dyme, Allison Gaudy

**Affiliations:** 1 Bristol Myers Squibb, Princeton, USA; 2 CenExel, Los Alamitos, USA; 3 CenExel, Atlanta, USA

## Abstract

**Aims:**

In pharmacokinetic studies of xanomeline and trospium chloride (KarXT), dosing with food reduced trospium bioavailability, potentially impacting tolerability. This study was designed to demonstrate taking KarXT with food after a slow up-titration is safe and tolerable.

**Methods:**

In pharmacokinetic studies of xanomeline and trospium chloride (KarXT), dosing with food reduced trospium bioavailability, potentially impacting tolerability. This study was designed to demonstrate taking KarXT with food after a slow up-titration is safe and tolerable.

**Results:**

During periods 1 and 2, 64.2% and 39.0% of participants reported ≥1 treatment-emergent adverse event (TEAE), respectively. The most common TEAEs (≥5%) in period 1 were nausea (22.5%), dyspepsia (15.6%), headache (15.0%), constipation (12.7%), and vomiting (11.6%); the incidence of all new onset TEAEs was less in period 2 when taken with food. All TEAEs were mild or moderate in intensity; none were serious. PANSS total score decreased over periods 1 and 2; Clinical Global Impression-Severity score decreased over period 1 with no further change over period 2. Dose-normalized area under the curve and maximum plasma concentration of trospium pooled from cohorts 1 and 2 decreased by 36% and 43%, respectively, when taken with food versus fasting. No clinically significant differences were observed in xanomeline exposure.

**Conclusion:**

After slow uptitration on an empty stomach, there was no increase in the incidence or severity of TEAEs when taking KarXT with food; efficacy was maintained.

